# T-cell epitope strength in WAP-T mouse mammary carcinomas is an important determinant in PD1/PD-L1 immune checkpoint blockade therapy

**DOI:** 10.18632/oncotarget.11620

**Published:** 2016-08-25

**Authors:** Michael Bruns, Jara Wanger, Udo Schumacher, Wolfgang Deppert

**Affiliations:** ^1^ Heinrich-Pette-Institute, Leibniz-Institute for Experimental Virology, Hamburg, Germany; ^2^ Institute for Anatomy and Experimental Morphology, University Medical Center Hamburg-Eppendorf (UKE), University of Hamburg, Hamburg, Germany; ^3^ Institute for Tumor Biology, University Medical Center Hamburg-Eppendorf (UKE), University of Hamburg, Hamburg, Germany; ^4^ Woldsenweg, Hamburg, Germany

**Keywords:** transgenic breast cancer mouse model, SV40 T-antigen, LCMV NP T-cell epitope, CTL response, differential response to immune checkpoint blockade therapy

## Abstract

Using the SV40 transgenic WAP-T/WAP-T_NP_ mouse models for mammary carcinomas, we compared the response to immune checkpoint blockade therapy in tumor mice expressing either SV40 T-antigen containing the LCMV NP-epitope (T-Ag_NP_ in WAP-T_NP_ mice), or the unmodified T-antigen (T-Ag in WAP-T mice). Specifically, we asked, whether the presence of the highly immunogenic NP-epitope in T-Ag_NP_ influences this response in comparison to the weakly immunogenic T-cell epitopes of T-Ag in WAP-T tumor mice. Treatment of WAP-T_NP_ tumor mice with either anti-PD1 or anti-PD-L1 antibodies led to tumor regression, with anti-PD-L1 treatment being more effective. However, tumors had fully re-appeared after 21 days, indicating that CTL exhaustion had been rapidly re-established. Surprisingly, the same treatment applied to WAP-T tumor mice resulted in a significantly prolonged period of tumor regression. We provide evidence that in contrast to the weak antigenic stimuli exerted by T-cell epitopes of T-Ag, the strong antigenic stimulus of the NP-epitope in T-Ag_NP_ has a dual effect: (i) a rapid generation of active NP-specific CTLs, accompanied (ii) by accelerated CTL exhaustion. Our data support the hypothesis that the immunogenicity of tumor antigen T-cell epitopes strongly influences the success of immune checkpoint blockade therapy.

## INTRODUCTION

Immune therapy is a promising approach for improving the treatment of cancer. However, the major obstacles in its successful application, the tumor-induced mechanisms that lead to immune-evasion, have not been satisfactorily resolved [1]. Analysis of the immune status of a given tumor entity and characterization of obstructed immune responses thus are crucial issues for the development of immune-therapeutic anti-cancer strategies [2]. Recently, novel immune therapy approaches aimed at inducing an immune checkpoint blockade, like treatment with anti-PD1 or anti-PD-L1 antibodies, have gained much interest, but have been successful only in a certain fraction of tumor patients [3], [4], [5], [6], [7]. Unfortunately, however, factors that influence the response to such approaches are not well understood so far. Due to the limited possibilities for analyzing the respective parameters in humans, suitable animal models should be of great value.

Our laboratory has developed inducible transgenic BALB/c mouse based models for triple-negative breast cancer (WAP-T and WAP-T_NP_ mice, respectively [8], [9], which allow the analysis of parameters controlling tumor-specific immune responses towards endogenously arising tumors in immune-competent mice. WAP-T and WAP-T_NP_ mice contain as transgene the Simian virus 40 (SV40) early gene region under control of the whey acidic protein (WAP) promoter. Induction of the transgene by lactotrophic hormones during late pregnancy and lactation leads to expression of the oncogenic SV40 early proteins T-antigen (T-Ag), small t-antigen, and 17kT protein specifically in epithelial cells of the mammary glands [8], [10]. Mammary carcinomas developing in WAP-T mice have been extensively characterized [10], [11], [12], [13], [14], and cross-species validation with the respective human tumor entities has confirmed that WAP-T and WAP-T_NP_ mice are suitable models for the respective human disease [8], [14].

In WAP-T_NP_ mice, the SV40 transgene additionally codes for the NP_118-126_-epitope contained within the nucleoprotein (NP) of lymphocytic choriomeningitis virus (LCMV), resulting in the expression of a chimeric T-Ag/NP protein (T-Ag_NP_). This allowed us to compare immune responses against the “weak” (i.e. low affinity) T-cell epitopes of SV40 T-Ag expressed by WAP-T mice with those against the “strong”, immune-dominant LCMV NP-epitope in T-Ag_NP_ expressed by WAP-T_NP_ mice. While immunization of WAP-T mice with SV40 did not induce a measurable immune response of cytotoxic T-lymphocytes (CTL), immunization of WAP-T_NP_ mice by LCMV infection induced a strong response which led to transient tumor cell elimination. Most intriguingly, WAP-T_NP_ mice mount an endogenous immune response (i.e. without immunization) against the LCMV NP-epitope, as elimination of CD8^+^ T-cells by anti-CD8^+^ antibodies or by irradiation accelerated tumor outgrowth in WAP-T_NP_ mice. WAP-T_NP_ tumor mice thus contain NP-epitope specific CD8^+^ T-cells, which, however, are only weakly active due to expression of the programmed death-1 protein (PD1). Consequently, treatment of WAP-T_NP_ tumor mice with anti-PD1 antibodies largely re-established their activity [9].

In this study we compared the response of WAP-T T1 tumor mice (expressing weakly immunogenic T-Ag epitopes) with that of WAP-T_NP_ NP8 tumor mice (additionally expressing the immune-dominant LCMV NP-epitope) to anti-PD1/PD-L1 immune checkpoint blockade therapy. Our data support the conclusion that the immunogenicity of T-cell epitopes strongly influences the duration of the anti-PD1/PD-L1 induced immune checkpoint blockade in WAP-T and WAP-T_NP_ tumor mice. Thus immunogenicity of tumor antigen T-cell epitopes appears to be an important factor in determining the success of immune checkpoint blockade therapies.

## RESULTS

### Heterogeneous PD-L1 expression in NP8 tumors

Inefficacy of CTLs in eliminating tumor cells largely results from the interaction of PD1 exhausted on CTLs with the PD1 ligand PD-L1 expressed on tumor cells and cells of the tumor microenvironment, [3], [6], [7], [15]. PD-L1 expression in NP8 mouse tumors is heterogeneous and seen only on a fraction of the tumor cells (Figure S1), as also described for PD-L1 expression in corresponding human mammary carcinomas [16], [17]. In addition, PD-L1 expression occurs in different patterns: PD-L1 expressing cells are either evenly distributed over the whole tumor area, either in a large (Figure S1A) or a small (Figure S1C) fraction of tumor cells, or occur in patches (Figure S1B). The enlarged image in Figure S1D demonstrates the cell surface expression of PD-L1 in NP8 tumors. A detailed analysis of PD-L1 expression in NP8 tumor mice will be described elsewhere (J. Wanger, M. Bruns, U. Schumacher, and W. Deppert, manuscript in preparation).

### Treatment of NP8 tumor mice with anti-PD1/PD-L1

**Figure 1:**
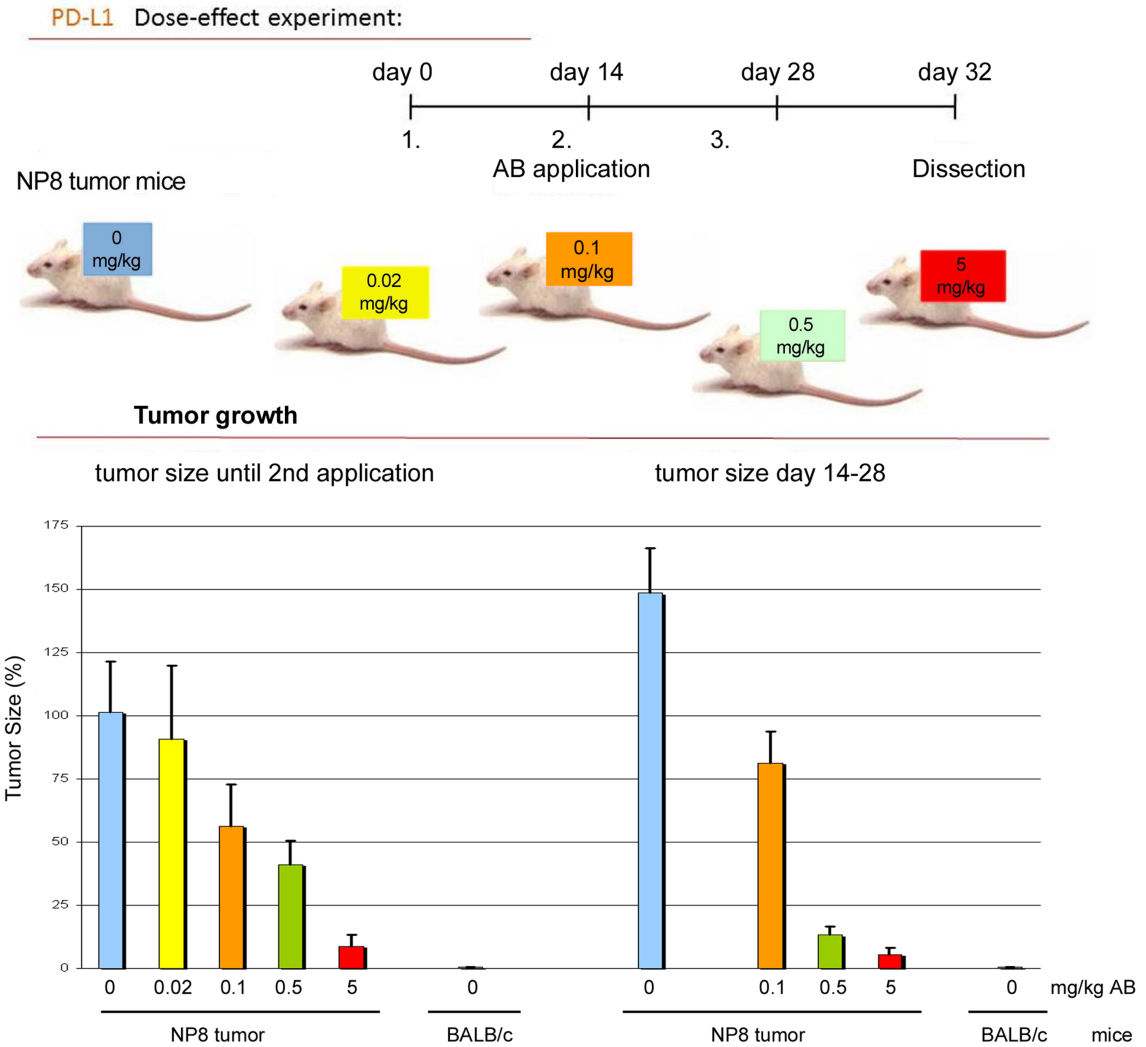
Dose dependent application of anti-PD-L1 antibodies to NP8 tumor mice NP8 tumor mice repeatedly received anti-PD-L1 antibodies in the intervals and concentrations shown in the experimental outline (upper panel). Thereafter we followed the inhibition of tumor growth under the influence of different amounts of antibodies within the times indicated; the stained columns in the graph below correspond to the differently labeled antibody doses presented in the outline above. Untreated NP8 mice (blue columns) and BALB/c mice (grey columns) served as positive and negative controls, respectively.

**Figure 2:**
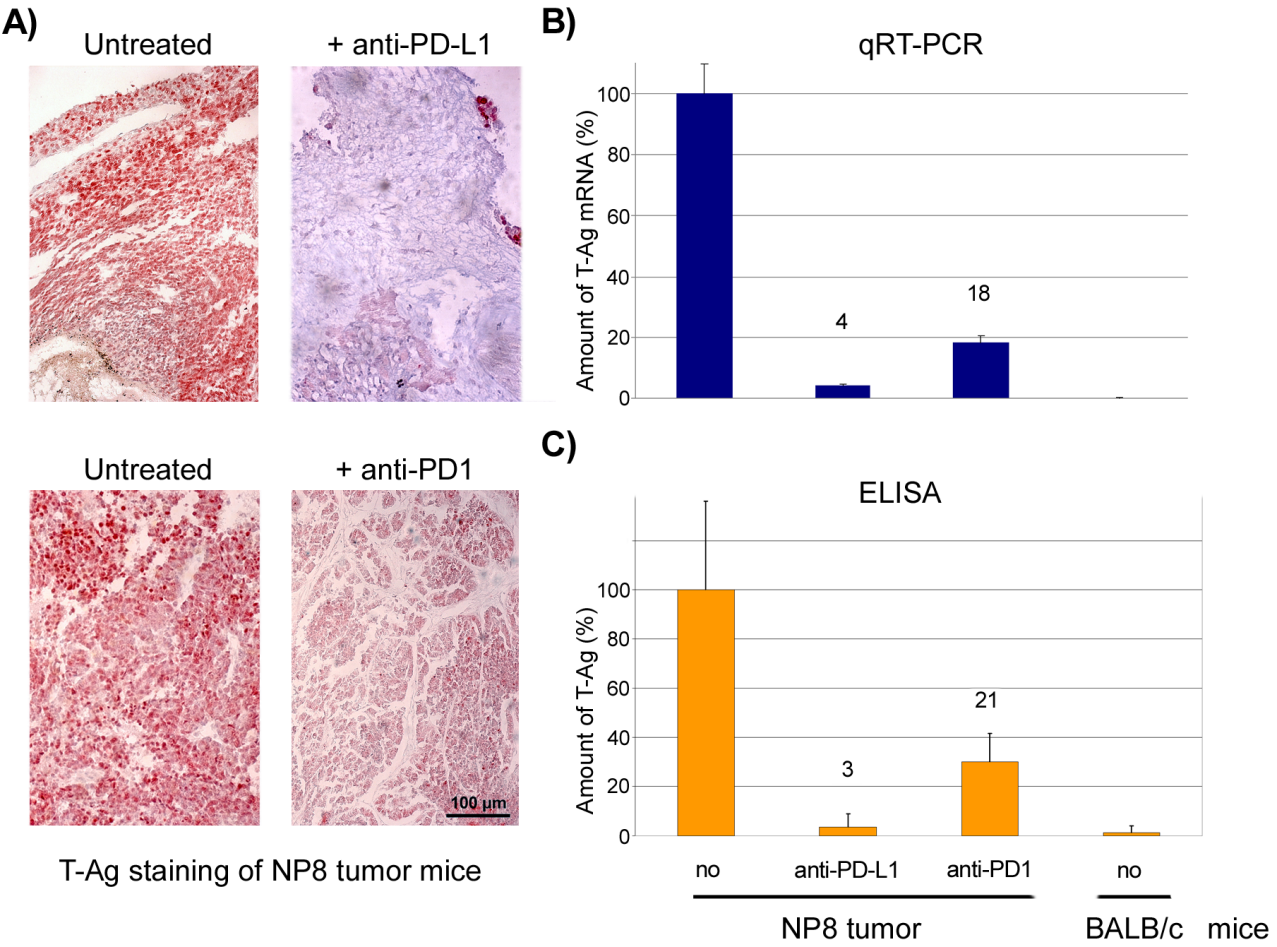
Responses of NP8 tumor mice to PD-L1 or PD1 antibody medication after 7 days **A.** NP8 tumor mice were treated with antibodies against PD-L1 or PD1 (0.5mg/kg) and tissue sections analyzed by histological staining with an anti-T-Ag antibody (magnifications are the same in all pictures; bar: 100μm). **B.** In parallel, T-Ag mRNA levels were measured by qRT-PCR and demonstrated a much stronger decline after the addition of anti-PD-L1 antibodies (4 %) than with anti-PD1 antibodies (18 %). **C.** A similar result was obtained by the analysis of the T-Ag protein levels determined by ELISA (anti-PD-L1 reduced the antigens to 3 %, anti-PD1 to 21 %). Untreated BALB/c mice served as controls in the PCR and ELISA experiments.

**Figure 3:**
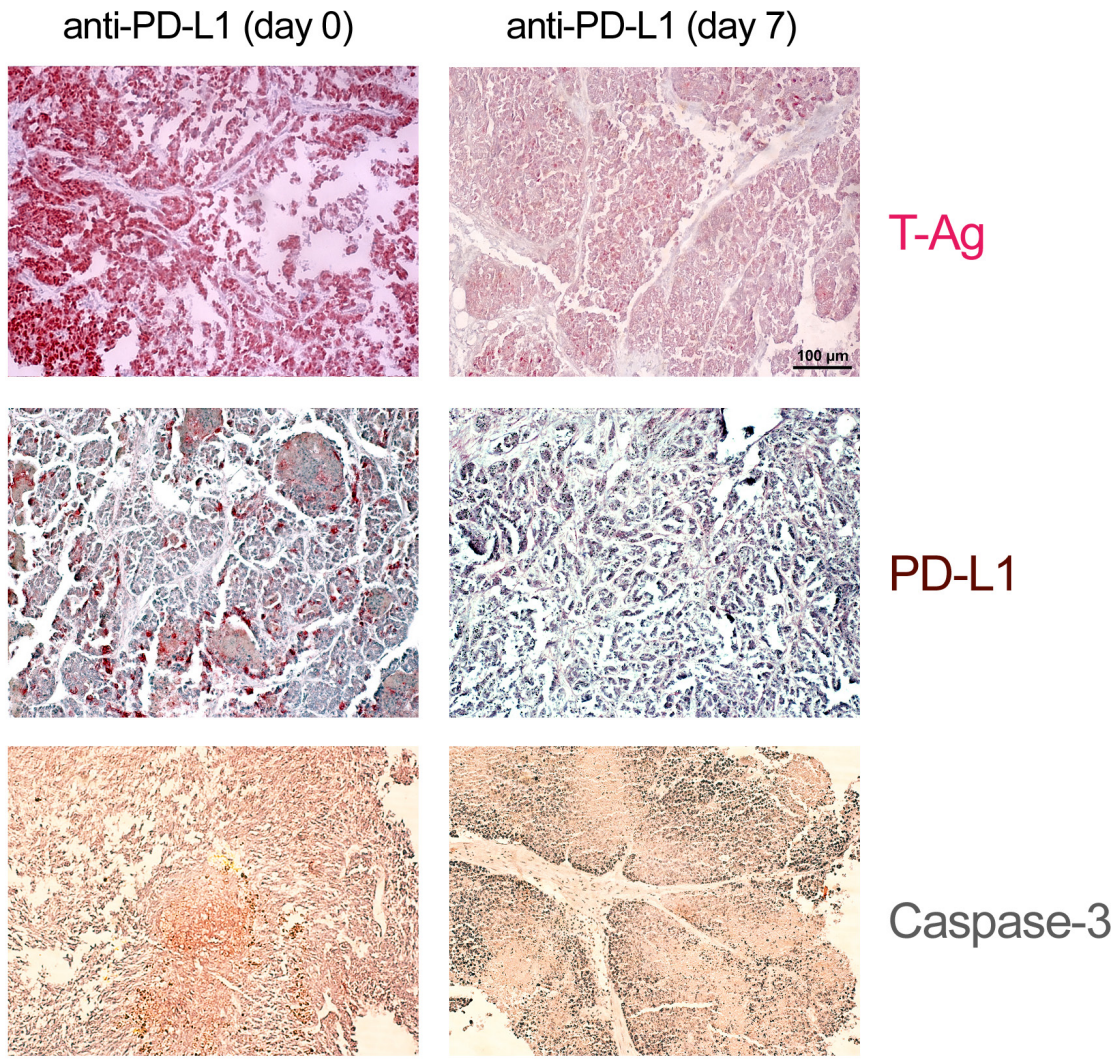
Histological evidence for tumor cell elimination after application of anti-PD-L1 antibodies NP8 tumor mice were treated as in described in Figure 2. Histologic examination of tumors of treated mice revealed massive reductions of T-Ag as well as of PD-L1 positive cells in comparison to that of untreated NP8 tumor mice, whereas at the same time the fraction of dying cells increased, as confirmed by the strongly enhanced presence of caspase-3 positive cells (magnifications are the same in all pictures; bar: 100μm).

**Figure 4:**
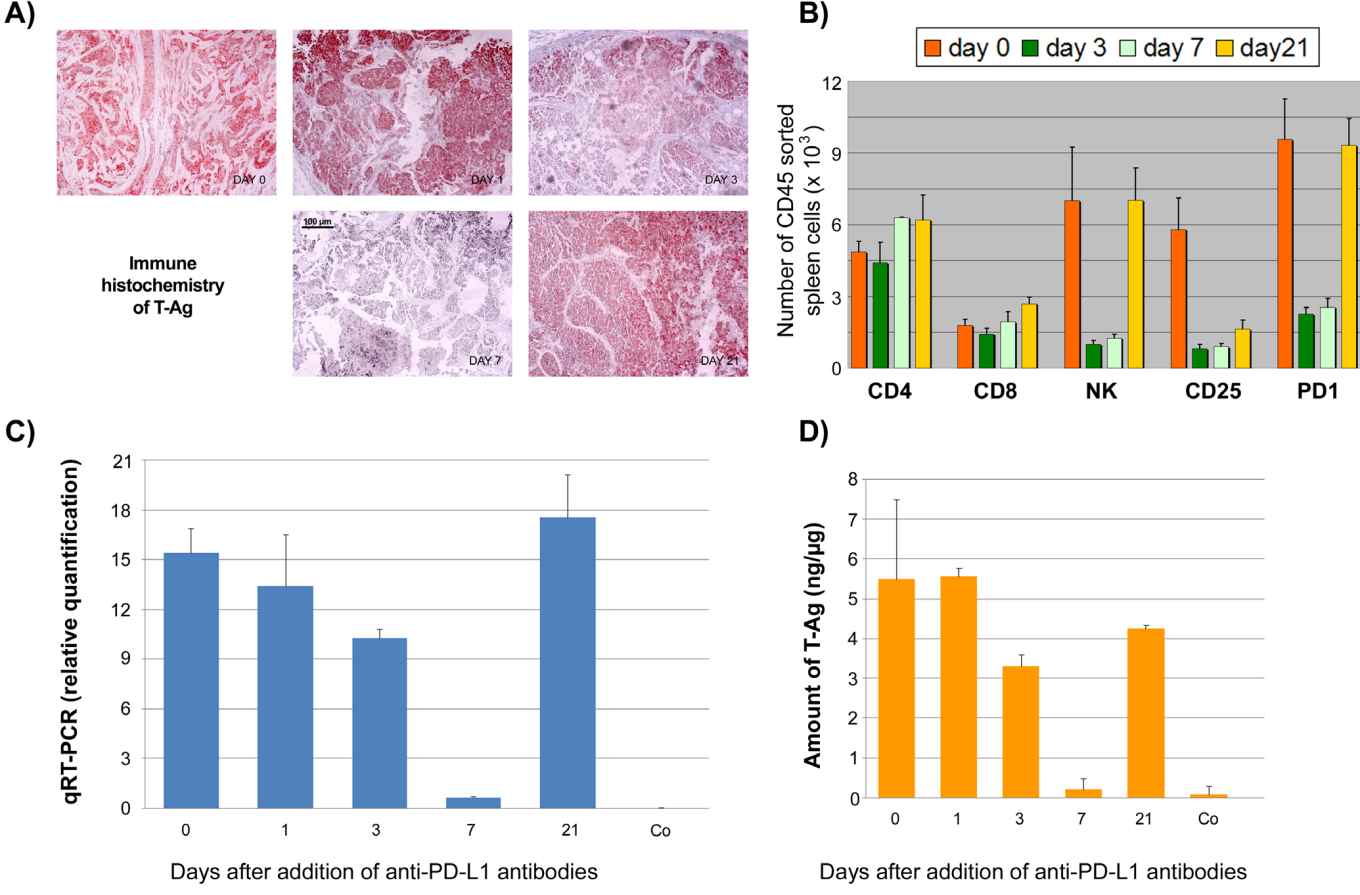
Time course analysis of the effects of anti-PD-L1 treatment of NP8 tumor mice NP8 tumor mice were treated as described in Figure 2. **A.** By immune-histochemistry, reduction of T-Ag positive cells starts on day 3 and is prominent on day 7. After 21 days tumor regrowth is almost complete (magnifications are the same in all pictures; bar: 100μm). **B.** Spleen lymphocytes were studied by FACS; while CD8+ cells and CD4+ cells remained largely unaffected with the exception of a slight increase for the former on day 21 and for the latter, somewhat more pronounced, on days 7 and 21, NK cells as well as CD25+ and PD1+ cells showed a dramatic decline beginning already at day 1 (not shown) and continuing over day 3 up to day 7 of treatment. On day 21 their levels had returned to original levels for NK as well as PD1+ cells, whereas at this time point increase in CD25+ cell level just had begun to rise again. **C.** Quantitative RT-PCR analysis revealed some reduction of T-Ag mRNA already on day 1 with a steady decrease until day 7; on day 21 T-Ag mRNA levels had increased to pre-treatment levels. **D.** Measurement of T-Ag protein levels by ELISA unveiled a drop of T-Ag beginning on day 3, reaching again its lowest quantity on day 7, and approaching pre-treatment levels on day 21.

We next compared the effects of anti-PD1 and anti-PD-L1 treatment of NP8 tumor mice (tumor size about 0.5 cm) as detailed in Materials and Methods. While the size of untreated tumors gradually increased, antibody treated tumor mice showed a dose-dependent reduction of tumor size after PD-L1 treatment (Figure 1). Figure 2, panel A shows the effect of anti-PD-L1 and of anti-PD1 treatments after 7 days as analyzed by immune-histochemical staining for T-Ag. The tumor areas appear virtually free of T-Ag_NP_ expressing cells. Quantitative evaluation of the same tumors for T-Ag mRNA (panel B) by qRT-PCR and for T-Ag protein (panel C) expression, however, revealed that anti-PD-L1 treatment was more effective than anti-PD1 treatment. We, therefore, in further experiments focused on anti-PD-L1 therapy. Immune-histochemical analysis of the effects of anti-PD-L1 therapy for NP8 tumors (Figure 3) shows the concomitant reduction of T-Ag_NP_ and of PD-L1 expressing cells. Staining for caspase-3 expression confirmed that the rapid destruction of tumor cells after anti-PD-L1 treatment is due to apoptosis. In a time course experiment, shown in Figure 4, we analyzed the effects of anti-PD-L1 treatment of NP8 tumor mice over a period of 21 days. Panel A reveals that already at day 3 a significant anti-tumor effect can be observed, as evidenced by the reduction in T-Ag_NP_ expressing tumor cells. At day 7 virtually no T-Ag_NP_ expressing cells are visible, but at day 21 the tumor had fully re-appeared. Loss of T-Ag_NP_ expression in tumors of anti-PD-L1 treated NP8 mice and its rapid re-growth was also verified by qRT-PCR for T-Ag mRNA in RNA derived from the same treated tumors (panel C) and by ELISA of T-Ag protein in tumor extracts (panel D). Tumor destruction and tumor re-growth are reflected in the quantitative analysis of the CD45^+^-lymphocyte composition in the spleens (panel B). There is a moderate increase of CD4^+^ T-cells, starting at day 7 continuing until day 21 after treatment, while CD8^+^ T-cells stay approximately constant with a moderate increase at day 21. Due to strong expression of PD-L1 on natural killer (NK) cells in tumor bearing mice [18], the fraction of NK cells is down significantly on days 3 and 7 after treatment, but has recovered on day 21. A similar reduction is seen in the fraction of CD25^+^ positive cells, encompassing regulatory T cells (T_reg_), which, however, did not accumulate again to their original level during the observation period. The most dramatic changes after treatment are observed in the PD1^+^ lymphocyte fraction. On days 3 and 7 after treatment, there is an about two-thirds drop, but the fraction of PD1^+^-lymphocytes had recovered completely by day 21, in accordance with tumor re-growth.

**Figure 5:**
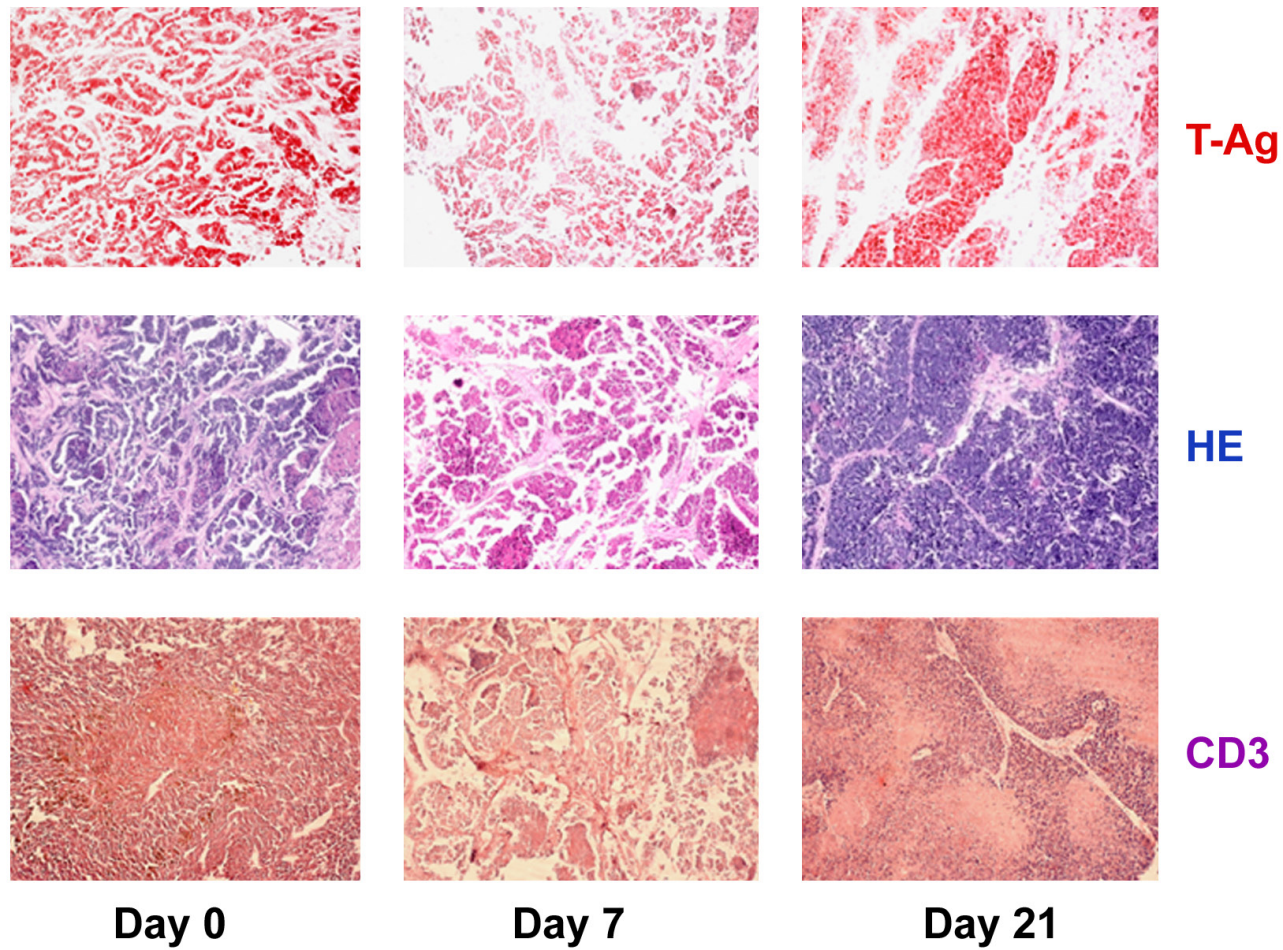
Changes in tumor-associated immune cells in tumors of anti-PD-L1 treated NP8 tumor mice on days 7 and 21, respectively NP8 tumor mice were treated as in described in Figure 2 and tumor sections analyzed at the time points indicated. Sections were either stained for T-Ag (red, upper row) or CD3^+^ cells (violet, lower row). HE stained samples (blue, middle row) served to detect all immune cells invading the cancerous tissues. While on day 7 residual tumor areas are virtually free of immune cells, a massive immune cell invasion, including invasion of CD3^+^ cells is visible on day 21.

Anti-PD-L1 treatment of NP8 tumor mice is also accompanied by dramatic quantitative changes in tumor-associated immune cells (Figure 5). Prior to treatment (day 0), tumors contain a substantial number of CD3^+^ tumor-associated immune cells (mostly consisting of CD8^+^ cells, but only few CD4^+^ cells, and of macrophages ([19], and unpublished]). On day 7 after treatment, i.e. at the height of the anti-PD-L1 reaction, tumors are almost devoid of immune cells, while on day 21, i.e. after tumor regrowth, tumors are full of immune cells again, indicative for ongoing strong immune reactions. A detailed analysis of immune cell composition in anti-PD-L1 treated NP8 tumors will be published elsewhere (J. Wanger, M. Bruns, U. Schumacher, W. Deppert, manuscript in preparation).

### Treatment of T1 tumor mice with anti-PD1/PD-L1

**Figure 6:**
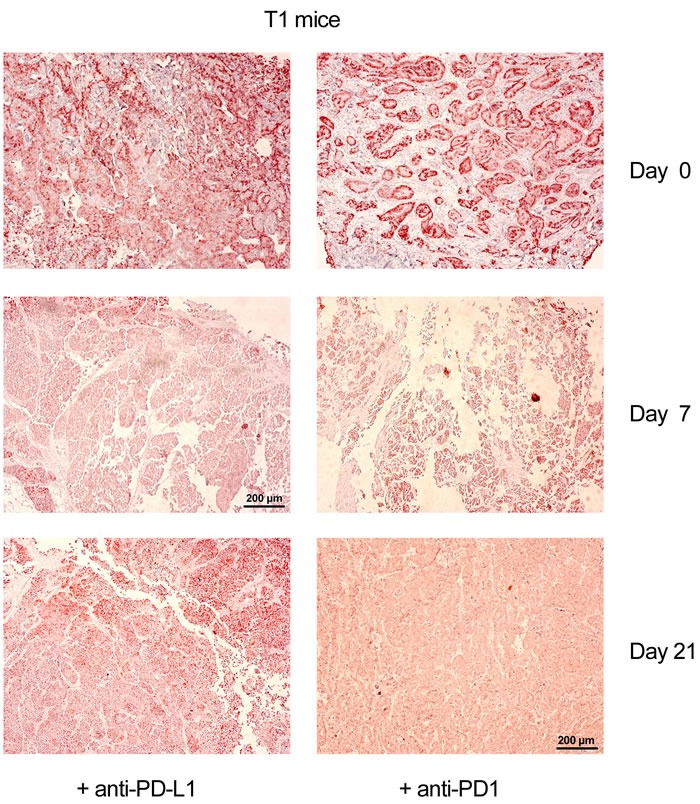
Anti-PD-L1 as well as anti-PD1 immune checkpoint control therapy is more efficient in T1 tumor mice lacking the NP T-cell epitope than in NP8 tumor mice Immune-histochemical analysis of tumor sections for T-Ag from T1 tumor mice, treated with anti-PD-L1 or with anti-PD1 antibodies (0.5mg/kg), showed for these mice a significantly extended period of tumor regression compared to treated NP8 mice, with no T-Ag expressing tumor cells up to 21 days (magnifications are the same in all pictures; bars: 200μm); T-Ag positive cells re-appeared in both cases about ten days later (not shown).

In NP8 tumor mice, the cytotoxic anti-tumor T-cell response is mainly, if not exclusively, directed against the LCMV NP-epitope expressed by the chimeric T-Ag_NP_ protein [9]. T1 derived tumors lack this epitope, and the weak, but measurable cytotoxic anti-tumor T-cell response is directed against the weak T-cell epitopes of SV40 T-Ag [20], [21], [22]. We next analyzed, whether the different antigenicity of T-cell epitopes expressed in NP8 and T1 derived tumors might influence the effects of immune checkpoint blockade therapy by anti-PD1/PD-L1 treatment. Therefore, we treated T1 tumor mice with anti-PD-L1 or anti-PD1 antibodies, respectively, as described above for the treatment of NP8 tumor mice. Figure 6 shows that, as in NP8 tumor mice, T1 tumors had efficiently regressed by day 7. Surprisingly, however, and in contrast to treated NP8 tumor mice, T1 tumors did not re-appear 21 days after treatment, thus leading to a significantly longer period of tumor regression up to day 31 (not shown). Quantitative comparison of treatment effects of NP8 and T1 mice by LCMV infection or anti-PD1/PD-L1 treatment (Figure S2) shows that LCMV infection is absolutely specific for NP8 mice and leads to virtually complete tumor regression after 21 days [9]. Anti-PD1/PD-L1 treatment leads to significant, though not complete tumor regression in NP8 and T1 mice after 7 days. While in NP8 mice the tumor has largely re-appeared after 21 days, no significant tumor regrowth is detectable in T1 mice within this time period.

**Figure 7:**
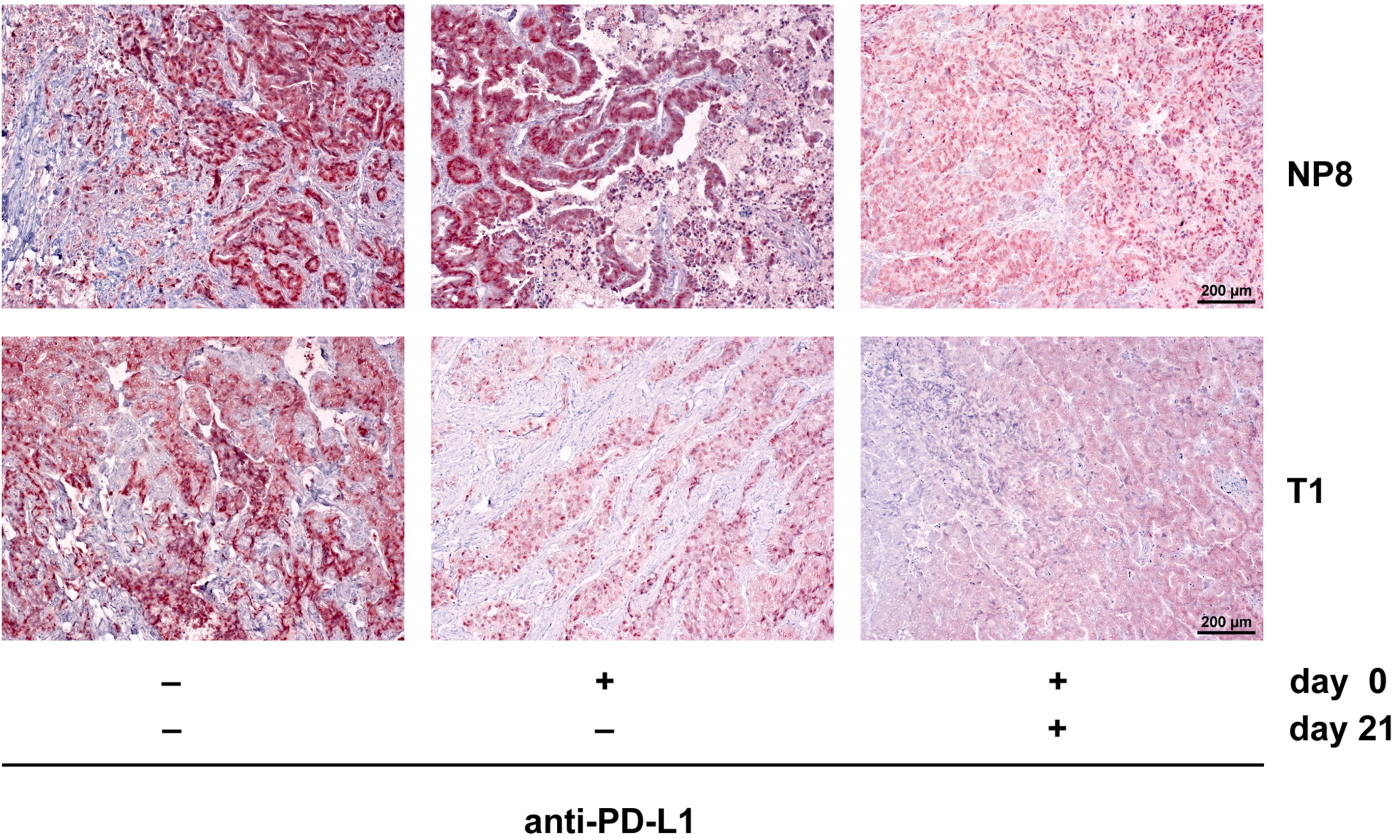
A second PD-L1 antibody treatment eliminates regrown tumors in anti-PD-L1 treated NP8 tumor mice and delays tumor regrowth in T1 tumor mice NP8 and T1 tumor mice were treated with a single dose of anti-PD-L1 antibodies (0.5mg/kg on day 0) and tumors harvested on day 28, or treated with a second dose on day 21 before harvest on day 28. Tumor regrowth was analyzed by immune-histochemical staining for T-Ag. After a single dose treatment, 28 days after treatment T-Ag staining in NP8 tumors was already as strong as in tumors of untreated mice, while a second dose on day 21 strongly reduced the number of T-Ag positive cells again. Tumors of T1 mice were virtually free of T-Ag positive cells after both treatments, indicating delayed tumor regrowth.

Tumor regrowth in both, NP8 and T1 mice after anti-PD-L1 treatment is due to re-establishment of T-cell exhaustion. This can be deduced from our finding that a second anti-PD-L1 treatment of NP8 again led to tumor regression and delayed tumor regrowth in T1 mice (Figure 7).

### The highly immunogenic NP T-cell epitope is a fast inducer of cytotoxic NP-specific T-cells, but also promotes T-cell exhaustion

As a possible explanation for the significant difference in the periods of tumor regression after anti-PD1/PD-L1 treatment of NP8 and T1 tumor mice, respectively, we considered that the high immunogenicity of the NP-epitope in T-Ag_NP_ of NP8 tumors might be responsible for the rapid abrogation of the immune checkpoint blockade. As endogenous tumors in T1 and NP8 mice are not suited to test this hypothesis, we resorted to our T1 and NP8 tumor derived tumor stem cell systems (G-2 and H8N8 cells, respectively). The G-2 as well as the H8N8 cell systems have been previously described and characterized in detail [23], [24], [25]. Both cell lines exhibit tumor stem cell properties, as 10 cells each suffice for tumor induction in NP8 mice after orthotopic transplantation. Transplanted tumors to a very large degree reflect endogenous tumors in terms of histology and molecular characteristics [23], [24]. G-2 and H8N8 derived tumors thus mimic endogenous tumors in T1 and NP8 tumor mice, respectively.

#### T-cell immunogenicity of G-2 and H8N8 tumor cell antigens in BALB/c or NP8 mice

To test for possible differences in the immunogenicity of tumor antigens in G-2 and H8N8 cells, we first compared the tumor take of G-2 and H8N8 cells after transplantation into BALB/c mice, i.e. into a close to isogenic host, except for lacking the SV40 transgene. Possible differences in tumor take then would indicate differences in the immunogenicity of T-antigen proteins either expressing the LCMV NP-epitope (T-Ag_NP_ in H8N8 cells) or not expressing this epitope (T-Ag in G-2 cells). As an isogenic control, we in parallel transplanted these cells into non-induced (virgin) NP8 mice.

Table 1 shows that in naïve BALB/c mice G-2 cells formed tumors in about 60% of the transplantations, while in NP8 mice 100% of the transplantations were successful. Treatment of BALB/c mice with low dose γ-irradiation [2 Gray (Gy)] prior to transplantation also allowed 100% tumor take. Still, tumor growth in untreated as well as in irradiated BALB/c mice was markedly slower than in NP8 mice (Figure S3; see also below). In contrast to transplanted G-2 cells, H8N8 cells never formed tumors in naïve BALB/c mice unless the mice were pre-treated with γ-irradiation (2 Gy). As expected, 100% of H8N8 cell transplantations were successful in NP8 mice. Like with G-2 cells, tumor growth of H8N8 cells in irradiated BALB/c mice was slower than in NP8 mice. To exclude the possibility that the differences in tumor take between G-2 and H8N8 cells were not related to the NP-epitope present in T-Ag_NP_, but might reflect intrinsic differences of the tumors of origin or of the cell lines themselves, we constructed G-2(Arm) cells (for details see Materials and Methods and Figure S4). G-2(Arm) cells are G-2 cells persistently infected with the replication-defective Armstrong strain of LCMV [26], [27], [28]. Due to defunct virus release, G-2(Arm) cells are loaded with NP (Figure S4). As expected, transplanted G-2(Arm) cells did not grow in naïve BALB/c mice. Also low dose irradiation (2 Gy) had to be repeated 7 days after transplantation to allow a 50% tumor take. However, anti-CD8 antibody pre-treatment of BALB/c mice allowed tumor outgrowth of transplanted G-2(Arm) cells. G-2(Arm) cell transplantations were 100% successful in NP8 mice, and G-2(Arm) cell induced tumors grew with similar kinetics as H8N8 cell induced tumors and as G-2(Arm) tumors in anti-CD8 treated BALB/c mice. This indicates that the somewhat stronger immune reaction against G-2(Arm) cells compared to H8N8 cells in BALB/c mice is due to the expression of large amounts of LCMV-NP in G-2(Arm) cells and not to additional factors, like e.g. residual virus release.

**Table 1:** Tumor growth after transplantations of WAP-T derived tumor cell lines into BALB/c or NP8 mice

Cell line	BALB/c				NP8uninduced
	untreated	1 × 2 Gy*	2 × 2 Gy**	anti-CD8	
G-2	4/6^a^ (40)***	9/9 (34)	n. d.	n. d.	5/5 (27)
H8N8	0/5 ( − )	5/5 (52)	n. d.	5/5 (34)	5/5 (34)
G-2(Arm)	0/8 ( − )	0/6 ( − )	3/6 (38)	4/5 (30)	7/7 (3)0

* Irradiation 1 day before transfer

** 1st irradiation 1 day before transfer, 2nd irradiation 7 days after transfer

*** Tumor bearing mice versus total number of mice; in brackets: means of days, until tumor size was about 1.5 cm (mice which had remained free of tumors for over 90 days were not used for calculation)

a A total of <30 G-2 cell transplantations were carried out during the course of our experiments, resulting in an average tumor take of ∼60%. The figure given here is from the parallel transplantation experiments shown in Table 1.

These data confirm that both, T-Ag and T-Ag_NP_, induce a cellular immune response in BALB/c mice. However, the immune response against the NP-epitope is much stronger than the immune response against the T-cell epitopes of T-Ag. The weaker immune response against T-Ag in BALB/c mice allows G-2 tumor cell outgrowth in about 60% of the transplantations, possibly by escape from destruction by immune cells and thus the possibility to establish a “tumor-friendly” microenvironment.

**Figure 8:**
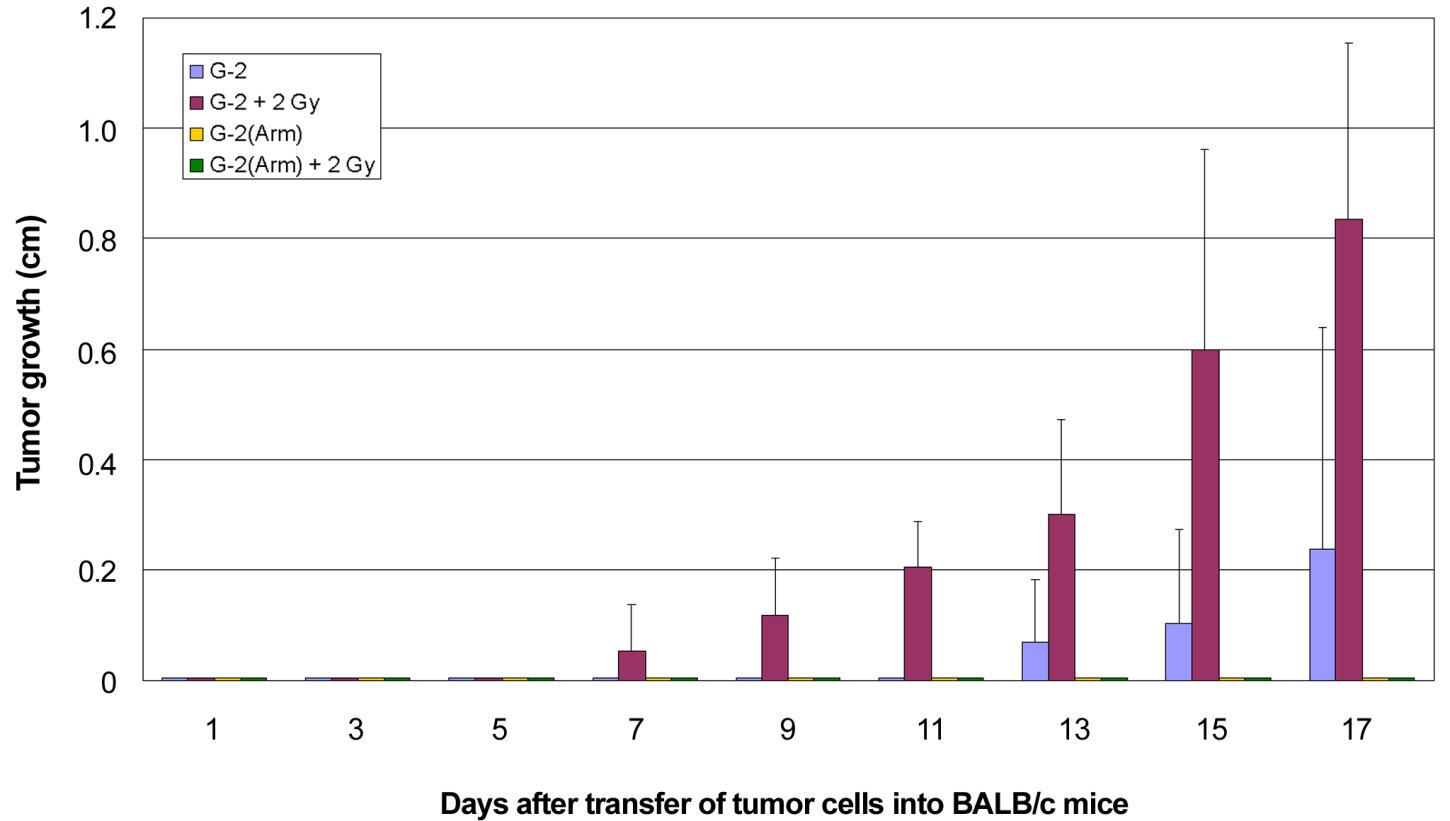
Growth kinetics of G-2 cell and G-2(Arm) cell induced tumors in BALB/c mice with or without γ-irradiation Enhanced and accelerated tumor growth was obtained after γ-irradiation of NP8 mice before transplantations of G-2 tumor cells (red columns) in comparison to untreated mice (blue columns). G-2 cells, persistently infected with an attenuated variant of LCMV, strain Armstrong, [G-2(Arm) cells] totally suppressed tumor outgrowth by an effective immune reaction (yellow columns); an intense immune reaction was also observed after 2 Gy γ-irradiation before transfer of G-2(Arm) cells (green columns), indicating inefficient elimination of CTLs by the irradiation.

With regard to the different immunogenicity of T-Ag derived T-cell epitopes in G-2 cells and the NP-epitope presented by G-2(Arm) cells it is interesting to compare the transplantations of G-2 and G-2(Arm) cells into untreated and into 2 Gy γ-irradiated BALB/c mice (Figure 8). Irradiation with 2 Gy allowed a 100% tumor take of transplanted G-2 cells and accelerated tumor growth by about 7 days compared to tumors growing in untreated BALB/c mice (Figure 8, red *versus* blue columns). However, tumor growth in irradiated BALB/c mice still was significantly slower than tumor growth of G-2 cells transplanted into NP8 mice (34 vs. 27 days, compare Table 1). This indicates that low dose irradiation dampened the CTL anti-tumor response, but did not completely suppress it. In contrast, no tumor outgrowth could be observed after transplantation of G-2(Arm) cells into 2 Gy irradiated BALB/c mice (Figure 8, green columns), even after longer times of observation (data not shown). We interpret this finding as to indicate that the NP-epitope presented by G-2(Arm) cells induced a strong T-cell response that could not be abolished by low dose irradiation, and that new CTLs had appeared before transplanted G-2(Arm) cells could establish a tumor. This interpretation is supported by our finding that a second 2 Gy dose of irradiation 7 days after G-2(Arm) cell transplantation, i.e. at the height of the NP-specific CTL response [29], at least allowed a 50% tumor take (Table 1). Tumor outgrowth, however, was still delayed by about 8 days compared to growth in anti-CD8 treated BALB/c mice and to growth in NP8 mice.

#### NP-epitope specific CTLs become rapidly exhausted

**Figure 9:**
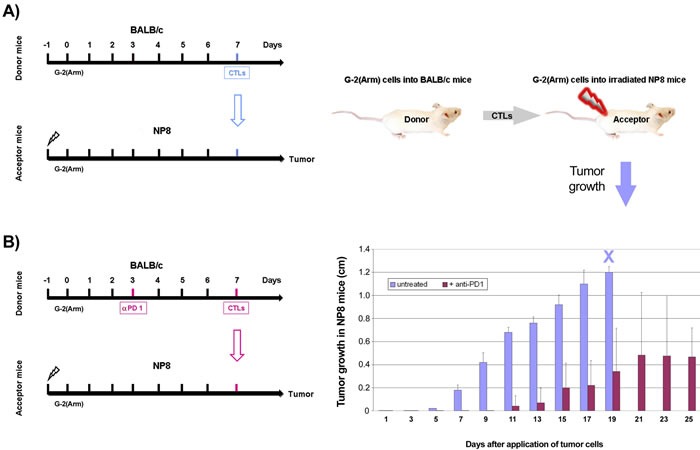
Adoptive transfer of NP-specific CTL from BALB/c donor mice into CTL depleted and G-2(Arm) cell transplanted NP8 acceptor mice to demonstrate NP-specific CTL exhaustion Left side: schemes of the experimental protocols, right side: graphic illustration of the experiments (above) and graph displaying the results (below). Results to experimental scheme **A.** After γ-irradiation of acceptor NP8 mice with 4 Gy one day before transplantation of 10^5^ G-2(Arm) cells, excessive tumor growth was observed (animals had to be killed on day 19 due to tumor size, blue X), which could not be suppressed by adoptive transfer of splenocytes from BALB/c donor mice which had been inoculated with 10^5^ G-2(Arm) 7 days before transfer (blue columns). Results to experimental scheme **B.** In a parallel experiment, performed under the same conditions, the donor mice were treated with anti-PD1 antibodies 4 days before the adoptive transfer of splenocytes, whereupon a powerful inhibition of tumor growth became evident (violet columns).

The high immunogenicity of the NP-epitope and the ensuing generation of highly active NP-specific CTLs as observed above in BALB/c mice does not seem to be compatible with the rapid abrogation of the immune checkpoint blockade in anti-PD1/PD-L1 treated NP8 tumor mice unless one assumes that the strong immunogenicity of the NP-epitope concomitantly also leads to fast CTL exhaustion. To address this question, we performed adoptive transfer experiments, as outlined in Figure 9, schemes A and B. As donor mice for the transfer of NP-specific CTLs we used splenocytes from BALB/c mice which had received a single dose of 10^5^ G-2(Arm) cells on day 0. In the experiment outlined in scheme A splenocytes were transferred on day 7 into NP8 acceptor mice. In the experiment outlined in scheme B, donor mice had received a single dose of anti-PD1 antibodies on day 3 after G-2(Arm) cell inoculation and splenocytes were transferred on day 7. CTL-depleted (4 Gy γ-irradiation on day -1) NP8 mice, transplanted on day 0 with G-2(Arm) tumor cells, served as acceptor mice. As shown in the graph in Figure 9, transfer of splenocytes obtained according to scheme A did not interfere with the growth of G-2(Arm) cells in NP8 acceptor mice. Thus NP-specific CTLs in the donor mice had become exhausted during the 7 days period after G-2(Arm) inoculation, and their transfer did not block tumor outgrowth of G-2(Arm) cells in NP8 acceptor mice. In contrast, splenocytes transferred according to scheme B led to a delayed and much slower growth of G-2(Arm) cells in NP8 acceptor mice, indicating that the anti-PD1 treatment of the donor mice 4 days before splenocyte transfer had reactivated exhausted NP-specific CTLs. One thus can conclude that the high immunogenicity of the LCMV NP-epitope on one hand induces a strong CTL response, but on the other hand also favors rapid exhaustion of these CTLs by PD1 expression.

Our data support the interpretation that the highly immunogenic NP-epitope presented by H8N8 tumor cells is responsible for the significantly shorter period of tumor regression in anti-PD1/PD-L1 treated NP8 tumor mice compared to T1 tumor mice that had received the same treatment. We thus conclude that the efficiency of an anti-PD1/PD-L1 immune checkpoint blockade therapy is strongly influenced by the strength of the T-cell epitope-specific antigenic stimulus exerted by tumor antigen(s). A strong antigenic stimulus will promote both, rapid generation of new CTLs, but also subsequent exhaustion of CTLs. In a treated tumor mouse expressing a strong tumor antigen T-cell epitope, exhaustion of CTLs will rapidly win over the generation of new active CTLs induced by residual tumor cells, thereby promoting rapid tumor re-growth.

## DISCUSSION

Conventional cancer therapies, like chemo-, radiation- and targeted therapies, aim at reducing tumor cell proliferation or inducing tumor cell death by interfering with tumor cell signaling, cell growth or cell division. In contrast, immunotherapies aim at boosting the anti-tumor immune response of the tumor patient. While conventional cancer immunotherapy strategies so far have had only very limited success [6], immune checkpoint blockade therapies are very promising new approaches in cancer therapy [3], [4], [6], [7], [30], [31]. Such therapies are designed to restore the patients’ own antitumor immune response that had been mitigated during the processes of tumor immune evasion. To date, immune checkpoint blockade therapies are performed against selected cancer entities using antibodies against the CTL-associated antigen-4 (CTLA-4), against the PD1 receptor, and against its ligand PD-L1, with success rates varying in the different cancer entities. To date, the most promising immune checkpoint blockade therapies seem to involve the PD1/PD-L1 axis, because of higher success rates and less adverse side effects (for details see reviews cited above).

A major and currently still unresolved problem of immune checkpoint blockade therapies is that the reasons underlying their success or failure are not well understood. It is assumed that expression levels of immune checkpoint proteins (rev. in [3] and [7]), as well as mutations enhancing the frequency of novel tumor antigen T-cell epitopes [32] play an important role. Our WAP-T/WAP-T_NP_ mouse models are suitable to address the respective pertinent questions.

In this study we focused on the influence of “weak” and “strong” tumor antigen T-cell epitopes on therapy outcome. WAP-T and WAP-T_NP_ tumors are histologically and molecularly extremely similar [10], but differ immunologically by the additional, immune-dominant LCMV NP-epitope expressed by the chimeric T-Ag_NP_ in WAP-T_NP_ tumors, which is not present in T-Ag of WAP-T tumors [8], [9]. We here analyzed the effects of anti-PD1/PD-L1 antibody therapies for the treatment of WAP-T/WAP-T_NP_ mouse mammary carcinomas. This approach is based on our previous observation that WAP-T_NP_ tumor mice contain LCMV NP-specific CD8^+^ T-cells which, however, are exhausted, as they displayed only limited activity in LCMV infected BALB/c mice [9]. Yet, activity could be re-established by treatment of WAP-T_NP_ tumor mice with anti-PD1 antibodies, suggesting a major role for the PD1/PD-L1 checkpoint axis in immune evasion of WAP-T_NP_ mammary carcinomas [9].

Analysis of PD-L1 expression in WAP-T/WAP-T_NP_ tumors showed that PD-L1 is strongly expressed in tumor tissue, not only by infiltrated immune cells, but also by epithelial tumor cells, and that it is located on the surface of tumor cells. As tumors in WAP-T mice model the triple-negative basal breast cancer (TNBC) subtype of human mammary carcinoma [13], [14], a relatively high expression of PD-L1 in WAP-T tumors is in line with PD-L1 expression in their human counterparts (rev. in [3] and [7].

When we compared the effects of anti-PD1 and anti-PD-L1 antibody treatments, respectively, on NP8 tumor regression, we observed that after both therapies tumors were no longer detectable visually (i.e. by size and immune-histochemical staining for T-Ag). However, molecular analyses for T-Ag mRNA and T-Ag protein revealed residual tumor cells and showed that anti-PD-L1 treatment was more effective than anti-PD1 treatment (below 5% residual tumor cells after anti-PD-L1 treatment compared to about 20% residual cells after anti-PD1 treatment). Although PD1 is also expressed on several other immune cells, it is mainly expressed on antigen-experienced T-cells, while PD-L1 is expressed on many cell types, including T- and other immune cells and the tumor cells themselves [6]. With regard to the higher efficacy of PD-L1 in our tumor system, it might be interesting to note that NK cells in our tumor mice highly express PD-L1, as is evident from the strong decrease of NK cells after anti-PD-L1 antibody treatment (see Figure 4B). Although tumor cell elimination primarily involves CD8^+^ T-cells, NK cells could play an additional role.

Due to its stronger anti-tumor effect and because of the reported lower side effects of the anti-PD-L1 antibody treatment (rev. in [6]), we concentrated on this approach. The strong anti-tumor effect of both treatments is due to tumor cell elimination, as can be deduced from the dose-dependent reduction of tumor size (Figure 1), mediated by enhanced tumor cell apoptosis, as shown in Figure 3.

It is assumed that anti-PD-L1 treatment leads to “reactivation” of exhausted T-cells by blocking the interaction of PD1 with PD-L1 [3], [33], [34]. However, the effects of anti-PD-L1 antibody treatment are not yet clear. The strong decrease in the number of CD3^+^ immune cells in tumors on day 7 after anti-PD-L1 treatment (Figure 5) might however suggest that anti-PD-L1 antibody treatment leads to apoptosis of PD-L1 expressing cells by antibody mediated cytotoxicity, as previously reported [35]. PD-L1 is also expressed on exhausted CD8^+^ T-cells, and the interaction of such cells with PD1 expressing T_reg_ cells is important for establishing and maintaining a chronic LCMV infection [36]. Considering that NP8 tumor mice should display a similar immune status as mice chronically infected with LCMV, we assume that the strong reduction of CD25^+^ T-cells, encompassing T_reg_ cells, as well as the dramatic drop in the fraction of PD1^+^-lymphocytes (Figure 4B) reflects the elimination of these cells by PD-L1 treatment. Our finding that we did not observe a measurable change in the overall CD8^+^ lymphocyte fraction after anti-PD-L1 antibody treatment supports our view that specifically exhausted CD8^+^ T-cells had been removed, as the fraction of NP-specific CD8^+^ T-cells (active or exhausted) is small (about 5%), and changes in this specific fraction are hardly detectable in analyses of the overall CD8^+^ T-cell fraction. Regardless, already a partial elimination of exhausted cells should shift the immunological balance between active and exhausted CTLs to the anti-tumor effector side, leading to tumor destruction [6].

To us the most surprising result was the finding that anti-PD1/PD-L1 treatment of T1 tumor mice resulted in a significantly longer period of tumor regression (up to 31 days compared to less than 14 days in NP8 tumor mice). Due to the close histological and molecular similarities of T1 and NP8 tumors, this difference can only be ascribed to the presence (in T-Ag_NP_ of NP8 tumors) or absence (in T-Ag of T1 tumors) of the highly immunogenic NP-epitope. We here provide evidence that the strong immunogenicity of this epitope indeed elicits a fast and strong CD8^+^ T-cell response (see Table 1 and Figure 8), but at the same also promotes rapid CD8^+^ T-cell exhaustion (see Figure 9). Although the respective experiments do not exactly model the actual events in NP8 tumor mice, they can explain the significantly shorter period of tumor regression in anti-PD1/PD-L1 treated NP8 compared to T1 tumor mice. During and after treatment, residual NP8 tumor cells will induce new active NP-specific CD8^+^ T-cells, which together with residual non-exhausted T-cells will kill most of the tumor cells. These cells, however, will rapidly become exhausted in the tumor-supporting microenvironment, thereby allowing tumor re-growth. In retrospective, the rapid exhaustion of CTLs induced by the NP-epitope explains, why induced WAP-T_NP_ mice develop tumors at all, which might have not been expected considering the strong immunogenicity of the T-Ag_NP_ tumor antigen.

Although the NP-epitope might be considered as an extreme example for the immunogenicity of a tumor antigen T-cell epitope, the data provided here and in our previous studies [9], [10] show that even such a strong T-cell epitope does not protect against tumor outgrowth, and that after induction tumors in WAP-T_NP_ and in WAP-T mice arise with similar frequency and kinetics. On the contrary, according to this study, its presence is actually detrimental for the success of an immune checkpoint blockade therapy.

On the other hand, the relatively good efficacy of the anti-PD-L1 treatment in T1 tumor mice supports the idea that tumors expressing weak tumor antigen T-cell epitopes respond much better to immune checkpoint blockade therapies because re-establishment of an exhausted status of CTLs against these epitopes takes much longer. In addition, the lack of an immune-dominant T-cell epitope will allow the simultaneous generation of CTLs against several tumor antigen T-cell epitopes, further aggravating the re-establishment of T-cell exhaustion after immune checkpoint blockade therapy. The latter is also supported by findings demonstrating that tumors expressing many mutated proteins respond significantly better to such therapies [32].

In summary, our data strongly support the view that immunogenicity of tumor antigen T-cell epitopes strongly influences the duration of an anti-PD1/PD-L1 induced immune checkpoint blockade, and thus is an important parameter in determining the outcome of an immune checkpoint blockade therapy. Next generation sequencing will allow the rapid identification of mutations in tumor proteins which might serve as tumor antigens, and the detailed analyses of tumor antigen T-cell epitopes and their relative strength in different HLA subtypes will further progress [37], [38]. It thus will become possible to select patient collectives amenable to immune checkpoint blockade therapy. Furthermore, it will become possible to increase the activity of checkpoint inhibition by combination therapies [39], and in combination with approaches additionally targeting oncogenic pathways associated with immune suppression and T cell exhaustion (see e.g. [40]). Such combination therapies might be extremely promising especially for targeting PD-L1 expression, as expression of this molecule is regulated by a variety of oncogenic pathways [31].

## MATERIALS AND METHODS

### Mice

Inbred BALB/c mice and BALB/c-based transgenic mice crossed into them were used for all investigations presented here and were held under specific pathogen-free conditions. From the various generated mouse lines available [8], [41] we selected the transgenic mice WAP-T_NP_ (NP8) and WAP-T (T1) containing either the BALB/c mouse specific CTL NP-epitope of LCMV within the SV 40 T-Ag (T-Ag_NP_ inNP8) or not (T-Ag in T1), respectively [9]. Both transgenic mouse lines are very similar in their characteristic of tumor formation [10]. If not stated otherwise, at least three mice per group were used in each experiment. All animals were kept under S1 conditions and handled according to German regulations for animal experimentations. All protocols had been approved by the Hamburg administration (Deppert/Bruns #13/06; Deppert/Wanger #20/10: “Früherkennung der Entstehung von Mammakarzinomen für immuntherapeutische MaΔnahmen im transgenen Mausmodell“).

### Treatments of mice

For anti-PD-L1/PD1 treatment experiments we used goat polyclonal B7-H1/PD-L1 or PD1 antibodies (R&D Systems). We first evaluated the dose-dependency for clearance of the exhausted immune status using the anti-PD-L1 antibody in NP8 mice. An intravenous (iv) dose of 0.5 mg of either antibody per kg mouse body weight was selected for subsequent studies, because it provided a strong protective reaction, see Figure 1 for anti-PD-L1 treatment; data for anti-PD1 treatment were similar (not shown).

For adoptive transfers mice were treated by sub-lethal γ-irradiations with a radiation dose of 1 × 2 Gy, considered as a weak treatment eliminating immunologically active cells for about one week, or alternatively either with 2 × 2 Gy (in a 7 days interval) or 1 × 4 Gy, both judged as strong radiation doses removing immune cells, especially CTLs, for about two to three weeks (personal experience). A similar extensive discharge of CTLs was obtained in mice, when they were treated with 400 μg of monoclonal anti-CD8 antibodies (mAbs) iv [42], [43]. In any case no impairment of health or variation in physiological behavior could be observed in mice undergoing such treatments.

### Transplantation of tumor cells

For transplantations experiments 1×10^5^ tumor cells were harvested from cultures and re-suspended in 50 μl of a 1:1 mixture of serum-free Dulbecco's modified Eagle's medium (DMEM) and BD Matrigel Matrix high concentration, growth factor reduced (BD Bioscience, San Jose, CA). Between 10 and 20 weeks old non-induced (virgin) female mice were anaesthetized by intraperitoneal injection of ketamine/xylazine. After an incision of about 5 mm into the skin the cell suspensions were injected into the left or right abdominal mammary gland (MG #3 or MG #6); carprofen (50 mg/ml) was applied as analgesic; the skin was closed by interrupted sutures after implantations.

### Propagation and cultivation of cells

Cultures of tumor-derived G-2 or H8N8 cancer cells served in particular for the precise calculations of tumor growth during therapeutic treatments. The procedures for their isolation and their growth characteristics were described elsewhere [10], [23], [24], [25]. The cells were maintained in DMEM containing 10% fetal calf serum and 2 mmol/l glutamine and cultivated at 37°C in a humidified atmosphere of 5% CO_2_.

For the estimation of the reactivity against the CTL-specific NP epitope within the epitope presenting NP8 mice new cancer cell lines were developed after infection of G-2 cells with the highly attenuated L(Arm) virus (mouse L cell-derived Armstrong strain of LCMV), followed by cultivations over 8 passages (16 days) as a prerequisite for transferring them, similarly as described for L(Arm), BHK(Arm), Vero(Arm), and MDCK(Arm), into the persistently infected tumor cell line G-2(Arm) [26]; due to the modification of the glycoprotein-precursor most of the attenuated L(Arm) virus remains cell-associated, whereupon nearly exclusively only the viral NP could be detected in high amounts within the modified cell lines [26], [28], [personal observations (see also Figure S4)].

### Immune histochemistry

Histopathology and analysis of transgene expression were essentially as already described [8]. In brief, mouse mammary tissues were fixed with 4% formaldehyde containing 1% acetic acid and embedded in paraffin. De-paraffinated sections were stained with hematoxylin and eosin. Immunostaining of SV40 T-Ag was performed on paraffin sections using a triple-step immune enzymatic method. De-paraffinated sections were reacted before antibody incubation with a commercial ‘target unmasking fluid’ (Dianova) in a microwave oven. Subsequently, sections were incubated overnight at 48°C with a 1 : 10,000 dilution of the polyclonal rabbit antiserum R15 against T-Ag [44]; in some cases the commercially available polyclonal goat antibody against PD-L1 (see above) or the polyclonal rabbit antibody against human/mouse active caspase-3 in dilutions of 1 : 1000 (R&D Systems) were applied according to the supplier's recommendations. Specifically bound primary antibody was in the case of anti T-Ag, detected using biotinylated anti-rabbit IgG and phosphatase-conjugated streptavidin from a commercial kit (Super Sensitive Detection System, Biogenex). Phosphatase enzyme activity was revealed with naphthol AS-BI phosphate in combination with hexazotized new fuchsine (Merck). For the primary antibodies against PD-L1 and caspase-3 mouse anti-goat as well as anti-rabbit peroxidase-conjugated antibodies were used and incubated with Histofine Simple Stain Mouse Max PO anti-goat or anti-rabbit (Nichirei, Amsterdam, NL) and detected by 3,3′-diamino benzidine chromogene; possible endogenous peroxidase activities in granulocytes, mast cells, and erythrocytes were blocked by pre-incubation with 30 % H_2_O_2_ solution in phophate buffered saline (PBS). Naïve rabbit serum served as control. Sections were slightly counterstained with hemalum. All photographs were taken by the Zeiss Axioplan2 imaging microscopic equipment with the camera ProgRes C12plus of Jenoptic using the Software ProgRes CapturePro 2.9.0.1.gy

### Protein measurement and detection of T-Ag in ELISA

The procedures were already described in more detail [45]. Briefly, the protein content was calculated using the Bio-Rad protein assay with the Bradford Reagent [46]. For the determination of the amounts of T-Ag an ELISA was carried out, where aliquots of the samples were adsorbed onto MaxiSorp Immunoplates (Nunc) for 2 h at room temperature. The detection of viral antigen was performed with the rabbit anti T-Ag antiserum R15 [44] followed by horseradish peroxidase-labeled goat anti-rabbit immunoglobulins (Medac).

### RNA extraction and measurement of mRNA for T-Ag

Isolation and extraction of RNA from frozen tissue samples were performed using the innuPrep RNA Mini Kit (Analytic Jena, Germany) and reverse transcribed with the High Capacity cDNA Reverse Transcription Kit (Applied Biosystems, Foster City, CA). In general 1 μg of the purified RNA was employed for the synthesis of cDNA. Quantitative real-time PCR (qRT-PCR) was performed with the Power SYBR Green PCR Mastermix in an ABI 7500 Fast thermal cycler (Applied Biosystems). Per 10 μl mix 5 ng of cDNAs and in concentrations of 100 nM the primer pairs SV40LTag-Q1 (sense: TCCTGGCTGTCTTCATCATC) as well as SV40LTag-Q2 (antisense: AGAAAGGTTCGACGCTGACAC) were used.

### FACS

Analysis of splenocytes was performed usually starting with 3 × 10^4^ cells per staining. Cells were washed and re-suspended in 100 μl PBS buffer; thereafter 1 μl of FITC- or PE-stained antibodies (BD Biosciences) were added according to the manufacturer's instructions. For the examination of the relevant cellular populations the following mAbs, all obtained from BD Biosciences, were used: rat anti-mouse CD8 as well as CD4, mouse anti-mouse NK1.1, hamster anti-mouse PD1, and mouse anti-CD25, and incubated in the dark for 2 h. In order to inhibit unspecific binding rat anti-mouse CD16/CD32 was included to each arrangement as Fc block. Appropriate rat anti-mouse IgG1, IgG2a, and IgG2b were introduced as isotype controls. Cells were resuspended in 500 μl FACS buffer (0.5 % FCS, 100 μM EDTA in PBS) and analyzed in a FACSAria I cell sorter (Becton Dickinson) with BD FACS Diva 5.1.3 software. Before analyses of the various immune cell compartments 1 × 10^5^ cells were sorted after the incubation of splenocytes with 1 μl anti-mouse CD45 antibodies for 2 h in the dark and then examined by FACS using the specific antibodies described above.

## SUPPLEMENTARY MATERIAL FIGURES



## ABBREVIATIONS

CTL: cytotoxic T lymphocyte
CTLA-4: CTL-associated antigen-4
DMEM: Dulbecco's modified Eagle's medium
ELISA: enzyme-linked immunosorbent assay
Gy: Gray; iv, intravenous
LCMV: lymphocytic chorio­meningitis virus
mAb: monoclonal antibody
NK: natural killer cell
NP: nucleoprotein of LCMV; NP8, WAP-T-NP8 mouse
PBS: phosphate buffered saline
PD1: programmed death-1 protein
PD-L1: ligand of PD1
qRT-PCR: quantitative real-time polymerase chain reaction
SV40: Simian virus 40
T1: WAP-T1 mouse
T-Ag: T-antigen of SV40
T-Ag_NP_: chimeric T-Ag/NP protein
TNBC: triple-negative basal breast cancer
T_reg_: regulatory T cell
WAP: whey acidic protein

## References

[1] Rabinovich GA, Gabrilovich D, Sotomayor EM (2007). Immunosuppressive strategies that are mediated by tumor cells. Annu Rev Immunol.

[2] Scott AM, Wolchok JD, Old LJ (2012). Antibody therapy of cancer. Nat Rev Cancer.

[3] Pardoll DM (2012). The blockade of immune checkpoints in cancer immunotherapy. Nat Rev Cancer.

[4] Pardoll D (2015). Cancer and the Immune System: Basic Concepts and Targets for Intervention. Semin Oncol.

[5] Momtaz P, Postow MA (2014). Immunologic checkpoints in cancer therapy: focus on the programmed death-1 (PD-1) receptor pathway. Pharmgenomics Pers Med.

[6] Adachi K, Tamada K (2015). Immune checkpoint blockade opens an avenue of cancer immunotherapy with a potent clinical efficacy. Cancer Sci.

[7] Bedognetti D, Maccalli C, Bader SB, Marincola FM, Seliger B (2016). Checkpoint Inhibitors and Their Application in Breast Cancer. Breast Care (Basel).

[8] Schulze-Garg C, Löhler J, Gocht A, Deppert W (2000). A transgenic mouse model for the ductal carcinoma in situ (DCIS) of the mammary gland. Oncogene.

[9] Bruns M, Wanger J, Utermohlen O, Deppert W (2015). An inducible transgenic mouse breast cancer model for the analysis of tumor antigen specific CD8+ T-cell responses. Oncotarget.

[10] Heinlein C, Krepulat F, Lohler J, Speidel D, Deppert W, Tolstonog GV (2008). Mutant p53(R270H) gain of function phenotype in a mouse model for oncogene-induced mammary carcinogenesis. Int J Cancer.

[11] Quante T, Wegwitz F, Abe J, Rossi A, Deppert W, Bohn W (2014). Aberrant Proliferation of Differentiating Alveolar Cells Induces Hyperplasia in Resting Mammary Glands of SV40-TAg Transgenic Mice. Front Oncol.

[12] Kumar M, Witt B, Knippschild U, Koch S, Meena JK, Heinlein C, Weise JM, Krepulat F, Kuchenbauer F, Iben S, Rudolph KL, Deppert W, Gunes C (2013). CEBP factors regulate telomerase reverse transcriptase promoter activity in whey acidic protein-T mice during mammary carcinogenesis. Int J Cancer.

[13] Otto B, Gruner K, Heinlein C, Wegwitz F, Nollau P, Heinlein C, Weise JM, Krepulat F, Kuchenbauer F, Iben S, Rudolph KL, Deppert W, Gunes C (2013). Low-grade and high-grade mammary carcinomas in WAP-T transgenic mice are independent entities distinguished by Met expression. Int J Cancer.

[14] Otto B, Streichert T, Wegwitz F, Gevensleben H, Klatschke K, Wagener C, Deppert W, Tolstonog GV (2013). Transcription factors link mouse WAP-T mammary tumors with human breast cancer. Int J Cancer.

[15] Keir ME, Butte MJ, Freeman GJ, Sharpe AH (2008). PD-1 and Its Ligands in Tolerance and Immunity. Annu Rev Immunol.

[16] Herbst RS, Soria JC, Kowanetz M, Fine GD, Hamid O, Gordon MS, Sosman JA, McDermott DF, Powderly JD, Gettinger SN, Kohrt HE, Horn L, Lawrence DP (2014). Predictive correlates of response to the anti-PD-L1 antibody MPDL3280A in cancer patients. Nature.

[17] Tumeh PC, Harview CL, Yearley JH, Shintaku IP, Taylor EJ, Robert L, Chmielowski B, Spasic M, Henry G, Ciobanu V, West AN, Carmona M, Kivork C (2014). PD-1 blockade induces responses by inhibiting adaptive immune resistance. Nature.

[18] Iraolagoitia XL, Spallanzani RG, Torres NI, Araya RE, Ziblat A, Domaica CI, Sierra JM, Nunez SY, Secchiari F, Gajewski TF, Zwirner NW, Fuertes MB (2016). NK Cells Restrain Spontaneous Antitumor CD8+ T Cell Priming through PD-1/PD-L1 Interactions with Dendritic Cells. J Immunol.

[19] Jannasch K, Wegwitz F, Lenfert E, Maenz C, Deppert W, Alves F (2015). Chemotherapy of WAP-T mouse mammary carcinomas aggravates tumor phenotype and enhances tumor cell dissemination. Int J Cancer.

[20] Schirmbeck R, Zerrahn J, Kuhrober A, Deppert W, Reimann J (1993). Immunization of mice with the N-terminal (1-272) fragment of simian virus 40 large T antigen (without adjuvants) specifically primes cytotoxic T lymphocytes. Eur J Immunol.

[21] Zerrahn J, Utermöhlen O, Warnecke G, Deppert W, Lehmann-Grube F (1996). Protective immunity in BALB/c mice against the simian virus 40-induced mKSA tumor resulting from injection of recombinant large T antigen. Requirement of CD8+ T lymphocytes. J Immunol.

[22] Utermohlen O, Schulze-Garg C, Warnecke G, Gugel R, Lohler J, Deppert W (2001). Simian virus 40 large- T-antigen-specific rejection of mKSA tumor cells in BALB/c mice is critically dependent on both strictly tumor-associated, tumor-specific CD8(+) cytotoxic T lymphocytes and CD4(+) T helper cells. J Virol.

[23] Wegwitz F, Kluth MA, Manz C, Otto B, Gruner K, Heinlein C, Kuhl M, Warnecke G, Schumacher U, Deppert W, Tolstonog GV (2010). Tumorigenic WAP-T mouse mammary carcinoma cells: a model for a self-reproducing homeostatic cancer cell system. PLoS One.

[24] Lenfert E, Maenz C, Heinlein C, Jannasch K, Schumacher U, Pantel K, Tolstonog GV, Deppert W, Wegwitz F (2015). Mutant p53 promotes epithelial-mesenchymal plasticity and enhances metastasis in mammary carcinomas of WAP-T mice. Int J Cancer.

[25] Maenz C, Lenfert E, Pantel K, Schumacher U, Deppert W, Wegwitz F (2015). Epithelial-mesenchymal plasticity is a decisive feature for the metastatic outgrowth of disseminated WAP-T mouse mammary carcinoma cells. BMC Cancer.

[26] Bruns M, Kratzberg T, Zeller W, Lehmann-Grube F (1990). Mode of replication of lymphocytic choriomeningitis virus in persistently infected cultivated mouse L cells. Virology.

[27] Bruns M, Dralle H, Gegin C (1997). Protection of mice by an attenuated variant against the wild-type lymphocytic choriomeningitis virus. Intervirology.

[28] Stocker C, Martinez Peralta L, Kratzberg T, Lohmann F, Bruns M (1994). Characterization of a virus variant produced by L cells persistently infected with lymphocytic choriomeningitis virus. J Gen Virol.

[29] Lehmann-Grube F, Moskophidis D, Lohler J (1998). Recovery from acute virus infection. Role of cytotoxic T lymphocytes in the elimination of lymphocytic choriomeningitis virus from spleens of mice. Ann N Y Acad Sci.

[30] Medina J, Zbaren P, Bradley PJ (2016). Management of Regional Metastases of Malignant Salivary Gland Neoplasms. Adv Otorhinolaryngol.

[31] Chen J, Ji T, Zhao J, Li G, Zhang J, Jin R, Liu J, Liu X, Liang X, Huang D, Xie A, Lin H, Cang Y (2016). Sorafenib-resistant hepatocellular carcinoma stratified by phosphorylated ERK activates PD-1 immune checkpoint. Oncotarget.

[32] Le DT, Uram JN, Wang H, Bartlett BR, Kemberling H, Eyring AD, Skora AD, Luber BS, Azad NS, Laheru D, Biedrzycki B, Donehower RC, Zaheer A (2015). PD-1 Blockade in Tumors with Mismatch-Repair Deficiency. N Engl J Med.

[33] Pardoll DM (2015). Distinct mechanisms of tumor resistance to NK killing: of mice and men. Immunity.

[34] Medina PJ, Adams VR (2016). PD-1 Pathway Inhibitors: Immuno-Oncology Agents for Restoring Antitumor Immune Responses. Pharmacotherapy.

[35] Fujii R, Friedman ER, Richards J, Tsang KY, Heery CR, Schlom J, Hodge JW (2016). Enhanced killing of chordoma cells by antibody-dependent cell-mediated cytotoxicity employing the novel anti-PD-L1 antibody avelumab. Oncotarget.

[36] Park HJ, Park JS, Jeong YH, Son J, Ban YH, Lee BH, Chen L, Chang J, Chung DH, Choi I, Ha SJ (2015). PD-1 upregulated on regulatory T cells during chronic virus infection enhances the suppression of CD8+ T cell immune response via the interaction with PD-L1 expressed on CD8+ T cells. J Immunol.

[37] Brooks SE, Bonney SA, Lee C, Publicover A, Khan G, Smits EL, Sigurdardottir D, Arno M, Li D, Mills KI, Pulford K, Banham AH, van Tendeloo V (2015). Application of the pMHC Array to Characterise Tumour Antigen Specific T Cell Populations in Leukaemia Patients at Disease Diagnosis. PLoS One.

[38] Caron E, Espona L, Kowalewski DJ, Schuster H, Ternette N, Alpizar A, Schittenhelm RB, Ramarathinam SH, Lindestam Arlehamn CS, Chiek Koh C, Gillet LC, Rabsteyn A, Navarro P (2015). An open-source computational and data resource to analyze digital maps of immunopeptidomes. Elife.

[39] Twyman-Saint Victor C, Rech AJ, Maity A, Rengan R, Pauken KE, Stelekati E, Benci JL, Xu B, Dada H, Odorizzi PM, Herati RS, Mansfield KD, Patsch D (2015). Radiation and dual checkpoint blockade activate non-redundant immune mechanisms in cancer. Nature.

[40] Spranger S, Gajewski TF (2016). Tumor-intrinsic oncogene pathways mediating immune avoidance. Oncoimmunology.

[41] Krepulat F, Lohler J, Heinlein C, Hermannstadter A, Tolstonog GV, Deppert W (2005). Epigenetic mechanisms affect mutant p53 transgene expression in WAP-mutp53 transgenic mice. Oncogene.

[42] Cobbold SP, Jayasuriya A, Nash A, Prospero TD, Waldmann H (1984). Therapy with monoclonal antibodies by elimination of T-cell subsets in vivo. Nature.

[43] Moskophidis D, Fang L, Gossmann J, Drjupin R, Löhler J, Bruns M, Lehmann-Grube F (1990). Virus-specific delayed-type hypersensitivity (DTH). Cells mediating lymphocytic choriomeningitis virus-specific DTH reaction in mice. J Immunol.

[44] Deppert W, Pates R (1979). Simian virus 40 specific proteins on surface of HeLa cells infected with adenovirus 2--SV40 hybrid virus Ad2+ND2. Nature.

[45] Maenz C, Loscher C, Iwanski A, Bruns M (2008). Inhibition of duck hepatitis B virus infection of liver cells by combined treatment with viral e antigen and carbohydrates. J Gen Virol.

[46] Bradford MM (1976). A rapid and sensitive method for the quantitation of microgram quantities of protein utilizing the principle of protein-dye binding. Anal Biochem.

