# Supplementary material for: T-cell epitope strength in WAP-T mouse mammary carcinomas is an important determinant in PD1/PD-L1 immune checkpoint blockade therapy
Source: Oncotarget. 2016 Aug 25;7(40):64543–59. doi: 10.18632/oncotarget.11620 (PMC5323098; doi:10.18632/oncotarget.11620)
Supplement: Supplementary file 1 [file oncotarget-07-64543-s001.pdf]

T-cell epitope strength in WAP-T mouse mammary carcinomas is an important determinant in PD1/PD-L1 immune checkpoint blockade therapy

Supplementary Material

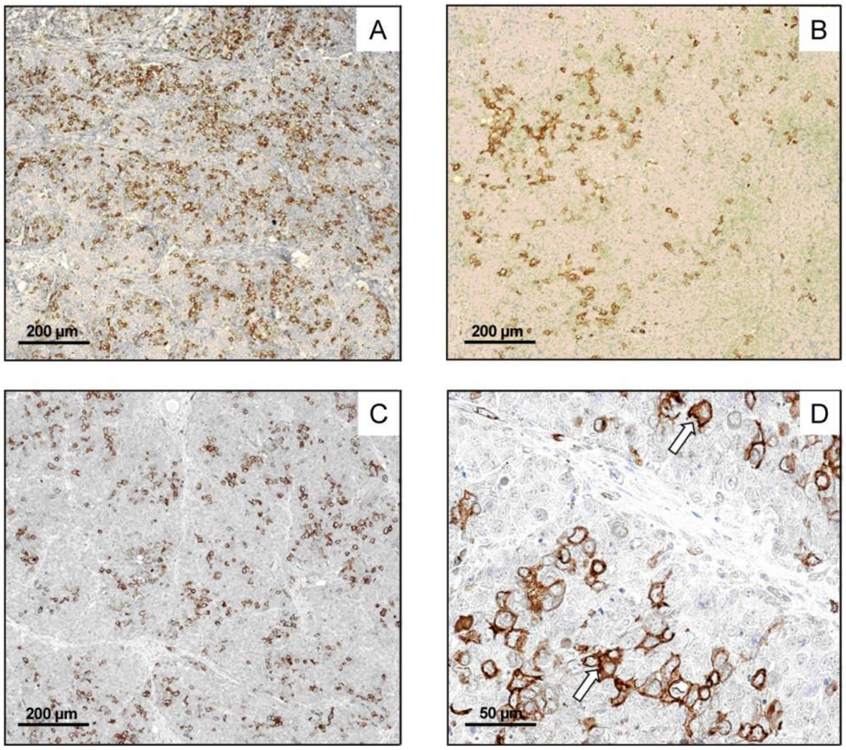

Fig. S1. Expression patterns and subcellular localization of PD-L1 in NP8 mamma carcinomas. In tissue sections positive cells are either spread over the whole tumor area (**A**) or located in more (**B**) or less isolated (**C**) groups (bars: 200µm); differences in the localization of the PD-L1 proteins within positive cells could be observed (**D**), where in most cases the cells exhibit an exclusive membranous staining, but is sometimes combined with a small circumference of cytoplasmic streaks (white arrows); the cells are in general of big, round, and pleomorphic shapes (bar: 50µm).

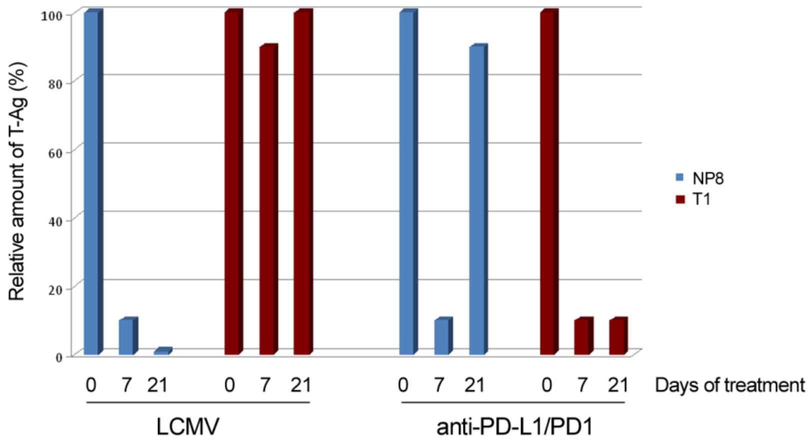

Fig. S2. Graph demonstrating the responses of tumor mice either presenting (NP8) or not presenting (T1) the strong LCMV NP-epitope. While infection with LCMV in NP8 mice eliminated tumor cells over a longer period, T1 tumor mice displayed only a marginal unspecific reaction as already described [9]. On the contrary, T1 mice showed an extended period of tumor regression compared to NP8 mice after anti-PD-L1/PD1 treatment.

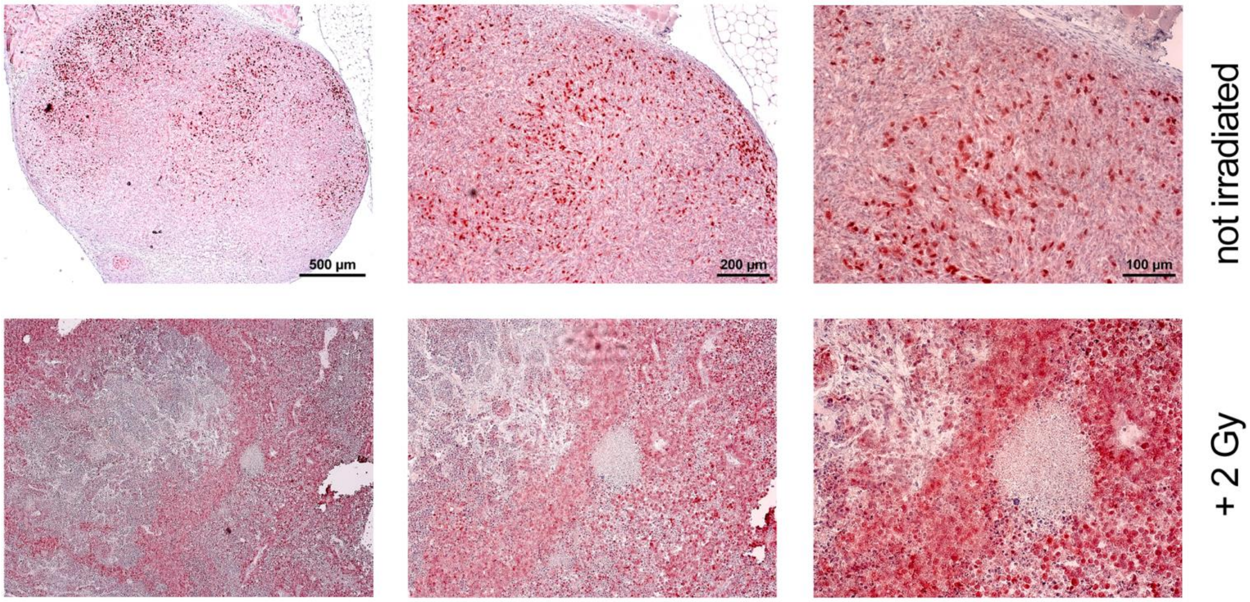

Fig. S3. Enhancement of tumor growth of transplanted G-2 cells after sublethal γ-irradiation of BALB/c mice. After transplantations of 10^5 G-2 tumor cells, BALB/c mice were either left untreated or were irradiated with 2 Gy; in the pictures depicted here one example of untreated as well as one example of treated tumors are shown in parallel after their growth

within 3 weeks in the magnifications of 500µm (left), 200µm (middle), and 100µm (right); the bars present in the upper row panels are also valid for the panels in the lower row.

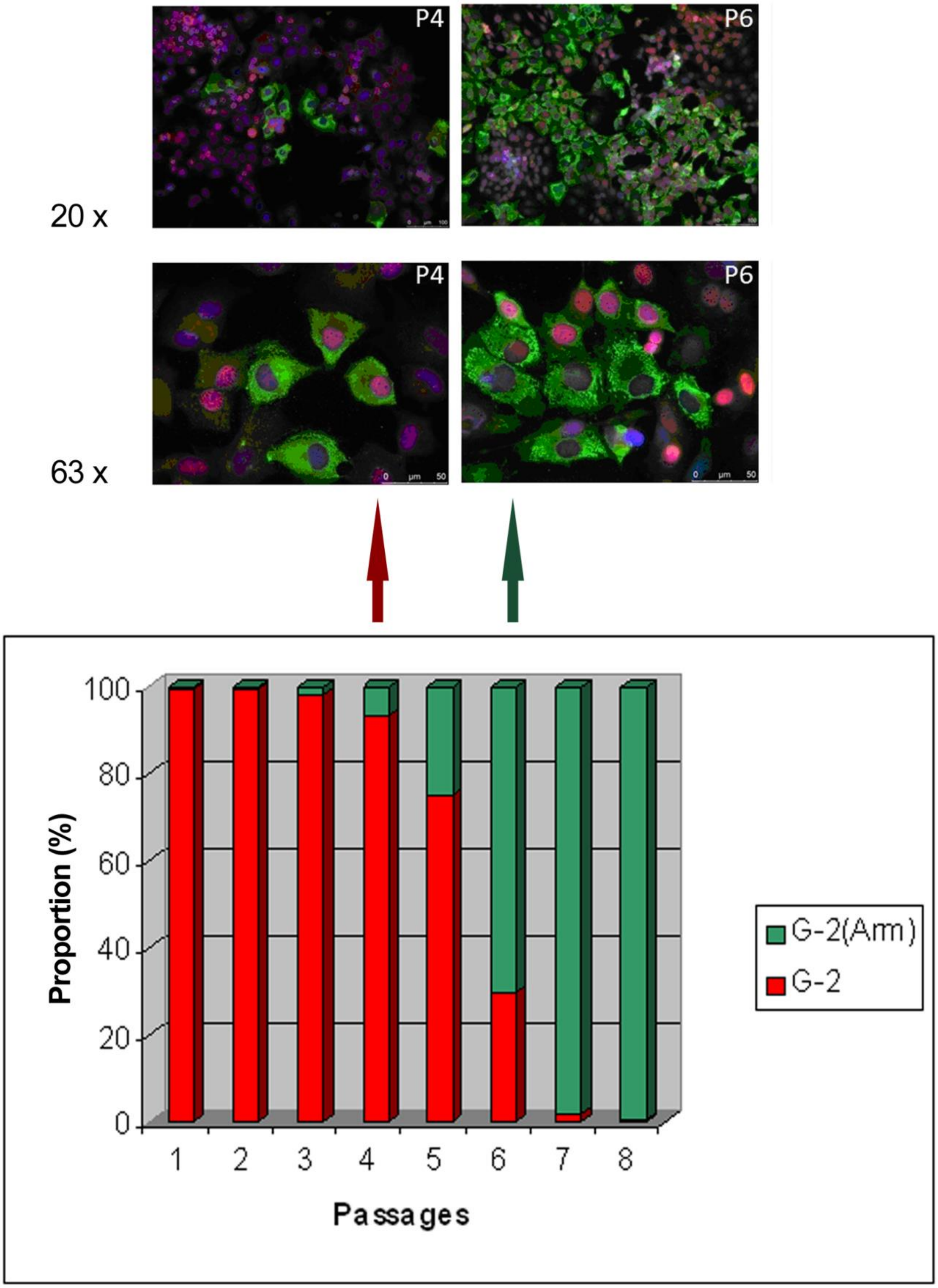

Fig. S4. Development of G-2(Arm) tumor cells. G-2 cells were infected using the attenuated LCMV Armstrong strain L(Arm) virus [26], [28]. Due to the high attenuation of the L(Arm) virus, as a rule 8 passages were necessary to transform G-2 cells permanently into G-2(Arm) cells,

finally resulting in cells, which expressed both, the NP of LCMV in the cytoplasm (green), and the T-Ag of SV40 in the nuclei (red); the two critical passages 4 and 6 representing the G-2 to G-2(Arm) switch were examined by immunofluorescence microscopy (above); the cells were plated on glass cover-slips, fixed with 4% paraformaldehyde in PBS, and immunostained using anti-T-Ag and anti-LCMV primary antibodies, followed by secondary fluorochrome-coupled antibodies (Invitrogen) according to the method described [23]. The graph (below) indicates the change in the proportion from G-2 to G-2(Arm), where the red parts of the columns symbolize the percentage of G-2 cells and the green part the percentage of G-2(Arm) cells.
